# A Cross-Sectional Study of Hematological Parameters in the Geriatric Population Using Peripheral Smear Examination and a Five-Part Cell Counter

**DOI:** 10.7759/cureus.57910

**Published:** 2024-04-09

**Authors:** Bhavinee Pathak, Sabiha Maimoon

**Affiliations:** 1 Pathology, NKP (Narendra Kumar Prasadrao) Salve Institute of Medical Sciences, Nagpur, IND

**Keywords:** 5-part cell counter, peripheral smear, complete blood count, hematology, geriatric

## Abstract

Background: "Healthy aging" is a major public health challenge, as the prevalence and incidence of diseases are much higher in older people. Among them, diabetes mellitus, hypertension (HTN), and cardiovascular illnesses are the most prevalent chronic ailments. A complete blood count test can give an overall picture of a patient's health status because abnormal counts might indicate the presence of many different types of disease. Using advanced hematology analyzers, a typical microscopic examination of a peripheral blood smear can yield vital information about the clinical state of the patient. The objective of the study was to investigate the hematological parameters in the elderly population in a tertiary care hospital, utilizing a five-part cell counter and a peripheral smear test.

Method: A cross-sectional study was conducted at a tertiary care institute on 188 patients aged 60 years and above attending the outpatient department for two years. The routine complete blood count and differential count were determined on the Siemens Advia 2120 analyzer. All the data were collected and entered into the data sheets. Appropriate statistical analysis was carried out to interpret the results.

Results: HTN, diabetes mellitus, cardiovascular disease (CVD), and generalized weakness were the most common conditions affecting our senior group. Decreased Hb was positively correlated with widespread weakness. Total leukocyte count (TLC) was found to be more prevalent in people with CVD and HTN. A sizable share of the elderly population who had diabetes mellitus and CVD showed an elevated red cell distribution width (RDW) percentage.

Conclusion: The results indicated a significant deviation from normal hematological parameters, which were associated with various health issues. These parametric associations can be used as risk indicators, which can help healthcare professionals ensure the good health of the geriatric population.

## Introduction

Given that older persons have a substantially greater prevalence and incidence of disease, "healthy aging" is a significant public health concern [1]. The "National Policy for Older Persons" defines the elderly as someone 60 years of age or older, and by 2030, India's elderly population is expected to reach 120 million [2].

Aging results in a physiological decline in bone marrow activity, perhaps in response to a drop in metabolic demand and tissue oxygen consumption. Aging is often linked to decreased activity and an increase in chronic inflammatory and cardiovascular diseases (CVDs). These factors affect not exclusively the formation of red blood cells but also plasma volume, increasing the likelihood of low hemoglobin (Hb) and hematocrit (HCT) levels [3]. The physiological aging process causes multiple health-related morbid conditions, such as anemia, CVD, hypertension (HTN), dyslipidemia, and diabetes. The decline in Hb, RBCs, and HCT levels that occurs more commonly in anemia is considered one of the most commonly seen conditions in elderly males and females. Anemia is caused by nutritional deficiency (vitamin B12, iron, and folate deficiency) in around one-third of patients [1,4]. Older adults possess a higher incidence of various risk factors for CVD such as diabetes, obesity, hyperlipidemia, sedentary lifestyle, and left ventricular hypertrophy [5].

The blood count is one of the most frequently used tests for health evaluation. A total blood count test can give an overview of the patient's general health state because abnormal blood counts can be an indication of the existence of many different diseases [6]. Using advanced hematology analyzers and a traditional microscopic examination of a peripheral blood smear might reveal vital details about a patient's clinical status [7].

With this background, the purpose of this study was to assess hematological parameters using a peripheral smear examination and a five-part cell counter in a geriatric population in a tertiary care hospital.

## Materials and methods

A cross-sectional study was conducted in the Department of Pathology and in the Central Pathology Laboratory at a tertiary care referral healthcare center over a period of two years. Approximately 188 participants aged 60 years and above who attended the outpatient department were included in the study after applying the simple random sampling technique. The commencement of the study was carried out only after approval from the institutional ethical board and informed consent forms were signed by the participants. The study was registered under the Clinical Trial Registry (CTRI/2022/11/047226). Patients with any malignancy and those with a known case of hematological disorder since childhood were excluded from the study. The study excluded patients with any malignancy or a known hematological disorder since childhood due to its focus on individuals without current malignancy. This allowed researchers to investigate hematological parameters in participants free from cancer-related factors and pre-existing conditions that may affect the blood profile since childhood. This exclusion ensured that the study's findings were more representative of the general elderly population, avoiding skewed results due to long-standing hematological disorders. Peripheral blood samples were collected in ethylene diamine tetraacetic acid (EDTA) anticoagulant tubes, and the routine complete blood count (CBC) and differential count were analyzed using a Siemens Advia 2120 five-part hematology analyzer (Siemens Healthineers, Erlangen, Germany). Fresh peripheral blood smears were stained with Leishman stain and examined under a binocular microscope. Complete blood count and scattergram were obtained from a five-part hematology analyzer.

Patterns of hematological derangements were observed in various geriatric patients using peripheral smear examination and automation. The demographic details along with the comorbidities of the patients were recorded in the case report form. The collected data were typed into an Excel sheet (Microsoft Corporation, Redmond, Washington) and were verified once per week on a computer to keep it secured. The data were kept confidential, and no name, phone number, or identity was disclosed. They were then reviewed, and the file was backed up. Data were evaluated periodically and typed into the Excel sheet for statistical analysis.

Data analysis

The collected data were fed into Microsoft Excel, and the data were analyzed in terms of percentage and frequency using IBM SPSS Statistics for Windows, Version 20 (Released 2011; IBM Corp., Armonk, New York). For qualitative data, the frequencies and percentages were calculated. The mean and standard deviation were calculated for the quantitative data.

## Results

**Figure 1:**
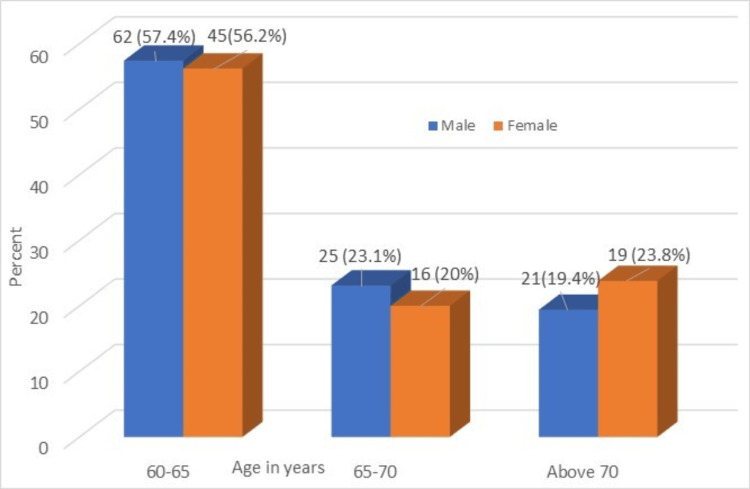
Age-wise distribution of the respondents

Age-wise distribution of the respondents is presented in Figure 1.

**Figure 2:**
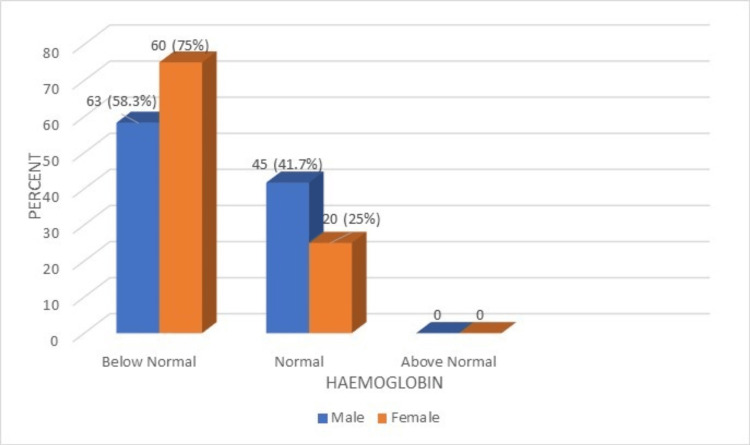
A comparison of hemoglobin percentage of the male and female geriatric population (normal range: 13-18 g/dL)

In the study, 56.2% of female participants and 57.4% of male participants enrolled were in the 60-65 years age group, and 23.1% of males and 20% of females were between the ages of 65 and 70 years. The remaining participants, comprising 19.4% of males and 23.8% of females, were over 70 years old. The comparison of the Hb percentage between male and female geriatric populations is shown in Figure 2.

**Figure 3:**
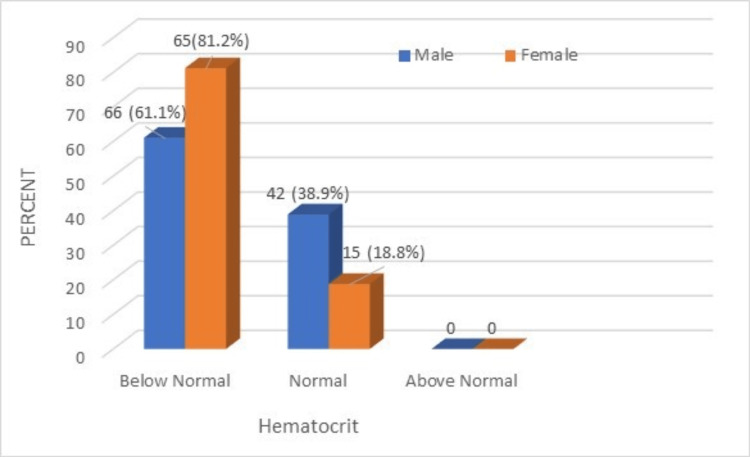
A comparison of HCT (PCV%) of male and female geriatric population (normal range: 40-54). HCT: hematocrit, PCV: packed cell volume.

The results of the study indicated that 41.7% of male respondents had normal Hb levels, while 58.3% of male respondents had lower Hb levels. Among the female respondents, 25% had normal Hb levels, and 75% had Hb counts below normal. Furthermore, the data revealed that the majority of elderly females had Hb counts that were below normal. The comparison of HCT (PCV%) between the male and female geriatric population is shown in Figure 3.

**Figure 4:**
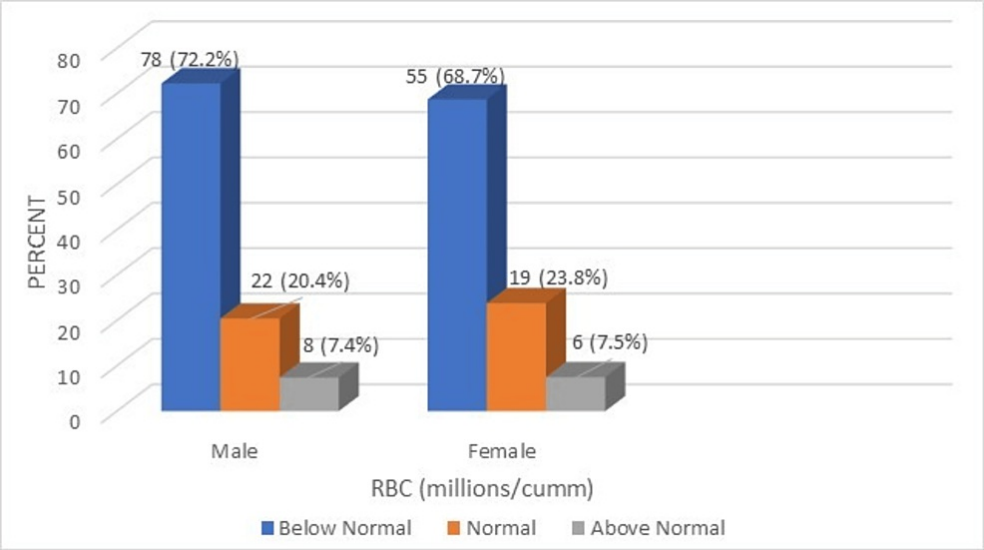
A comparison of RBC levels (millions/cubic millimeter) of the male and female geriatric population (normal range: 4.74-5.49).

Among males, 61.1% of respondents had below-normal HCT% levels. Among females, 81.2% of respondents had below-normal HCT% levels. This indicates that most of the male and female geriatric population had below-normal HCT% levels. The comparison of RBC counts between the male and female geriatric population is shown in Figure 4.

**Figure 5:**
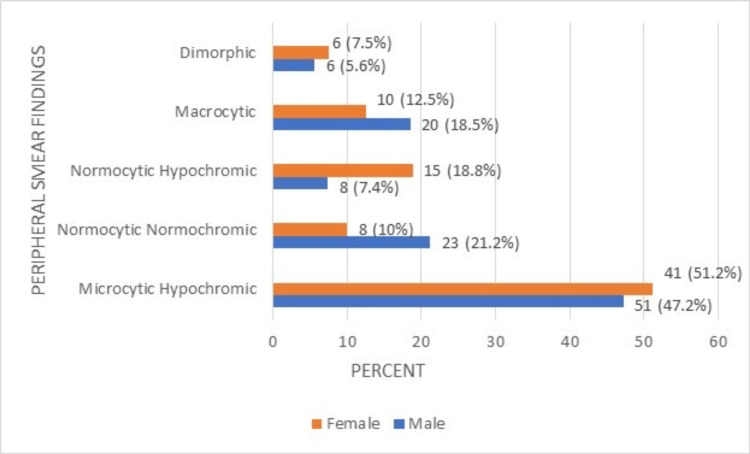
Comparison of peripheral smear findings of RBC of the male and female geriatric population.

The RBC count among males and females was found to be lower in 72.2% and 68.7% of respondents, respectively. This indicates that most of the male and female geriatric population had below-normal RBC counts. A comparison of peripheral smear findings of RBC of the male and female geriatric population is referenced in Figure 5.

**Figure 6:**
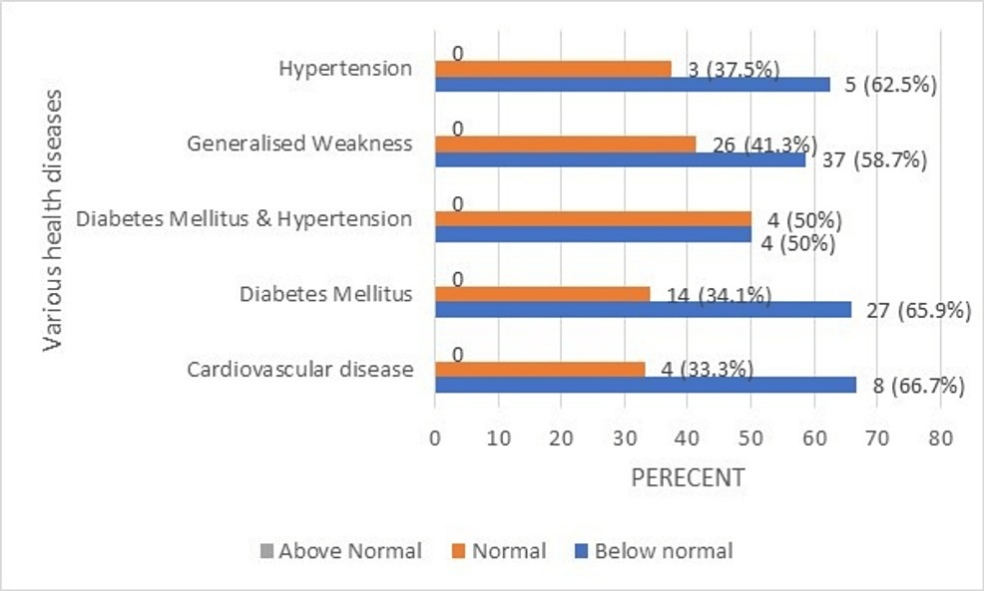
Hemoglobin percentage of the geriatric population having various health diseases (normal range: 13-18 g/dL).

The study findings indicated that the majority of the male (47.2%) and female respondents (51.2%) had microcytic hypochromic anemia. The Hb percentage of the geriatric population with various health issues is referenced in Figure 6.

**Figure 7:**
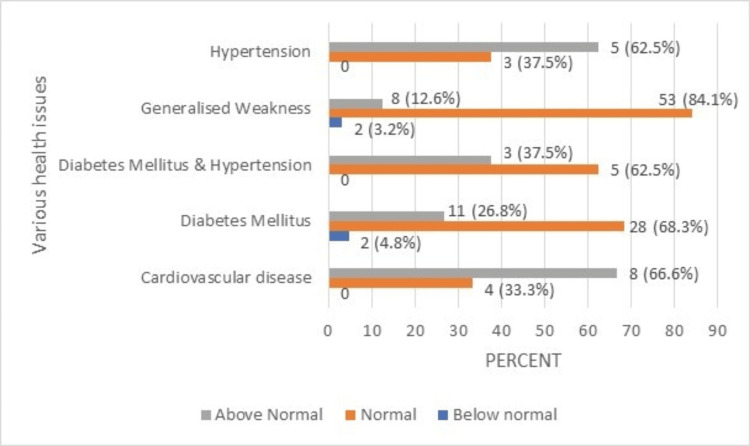
TLC percentage (normal range: 4000-11000/µL) of the geriatric population having various health issues. TLC: total leukocyte count.

Based on the findings, it was apparent that the majority of elderly individuals with a variety of illnesses, including HTN, diabetes mellitus, CVD, and generalized weakness, had Hb levels below normal. The total leukocyte count (TLC) percentage of the geriatric population with various health issues is shown in Figure 7.

**Figure 8:**
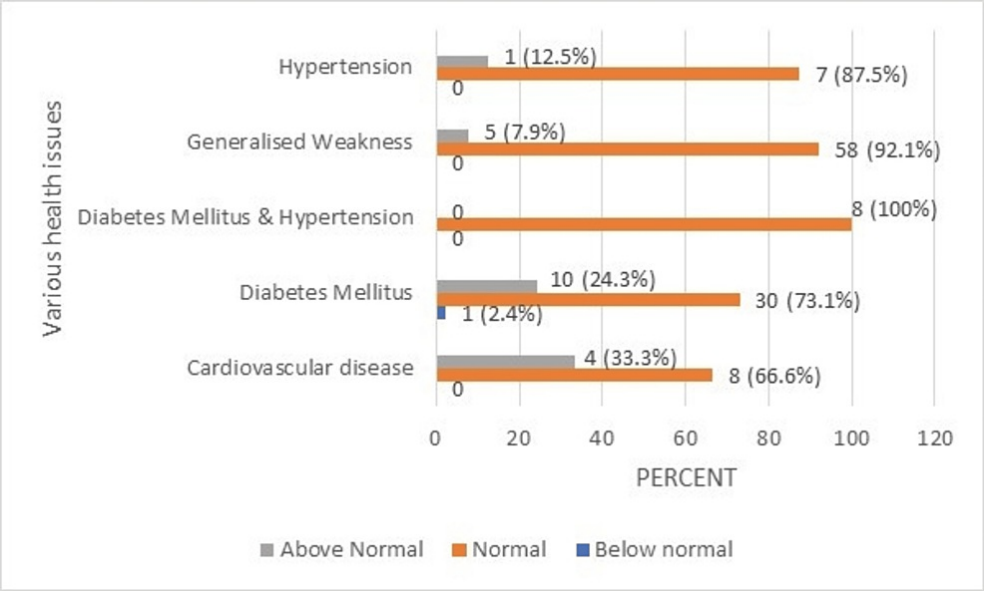
RDW percentage (normal range: 11.5-16.5) of the geriatric population having various health issues. RDW: red cell distribution width.

From the findings presented, it was clear that a large proportion of elderly individuals with an array of diseases, including diabetes mellitus, diabetes mellitus and HTN, and generalized weakness, have normal TLC percentages. In contrast, older individuals with CVD and HTN have TLC percentages that are above normal. The red cell distribution width (RDW) percentage of the geriatric population with various health issues is shown in Figure 8.

In all, 33.3% of the respondents with CVD had above-normal RDW. Furthermore, 24.3% of the respondents with diabetes mellitus also had above-normal RDW levels.

## Discussion

One of the most prevalent procedures for evaluating a patient's health is hematological or blood tests. These tests are crucial for detecting irregular blood test results and assisting doctors in managing patients with blood disorders, a wide range of illnesses, and life-threatening conditions. However, despite its widespread clinical application, significance, and almost universal accessibility, there is still uncertainty about the right reference intervals for the elderly in the world’s aging population. The quality of care needed for older adults is still lacking and represents a threat to the nation’s healthcare system. For the better management of such vulnerable patients, a large amount of human resources with better diagnostic and treatment modalities is crucial [8]. This study had age-specific criteria and involved only the sample population above 60 years of age to assess hematology analytes or parameters such as Hb, WBC, RBC, and platelets using specific examinations. Thereby, this research noted that the male subjects' participation was more and fell in the range of 60 and 65 years of age; and also revealed a notably high percentage of participants had below normal levels of Hb levels (58.7% of males, 74.7% of females), HCT levels (60.6% of males, 81% of females), and RBC counts (72.5% of males, 68.4% of females). Additionally, the analysis of blood films revealed a remarkably high prevalence of hypochromic microcytic RBCs (50.6% in males and 47.7% in females), which was suggestive of iron deficiency anemia and chronic illness. Low protein intake, nutritional inadequacies, myelodysplastic syndrome, gastrointestinal bleeding, and early hospitalization have all been linked to anemia according to other studies with comparable findings [9,10]. Hence, we conclude that there is a positive association between low Hb levels and generalized weakness.

Another parameter tested was the white blood cells or leukocytes, which are biomarkers for the detection and risk of infection in the blood. The TLC in this study was normal, in contrast to earlier studies that revealed a slightly lower count [11,12]. We found that the TLC was high in patients with CVD and HTN, as geriatric populations are prone to such illnesses including generalized weakness. High TLC counts in CVD and HTN patients may be due to vessel plugging, abnormal leukocyte aggregation, association with atherosclerotic risk factors, effects on blood flow, and proliferation of smooth muscle cells within the vessel wall [13,14]. Abnormal and deranged TLC count correlates with HTN, body mass index, and blood triglyceride levels [15]. Madjid and Fatemi also found that a high WBC level is a significant and independent predictor of coronary risk [6].

Red cell distribution width (RDW) is a calculated indicator of erythrocyte heterogeneity that reflects variation in circulating RBC size, and conditions such as inflammation, erythropoietin resistance, hemolysis, deranged Hb may cause changes in shape and size of RBCs [16-19]. Generally, a raised RDW is indicative of a variation in the size of RBCs beyond what is considered normal. Moreover, a raised RDW could be a sign of anemia and can be seen in chronic inflammation and increased oxidative stress [20]. This study displayed immoderate levels of RDW in the populace, similarly seen in other studies' findings, and linked with a higher incidence of death in CVD and diabetic patients respectively [13,20].

The presence of microcytic hypochromic conditions was seen in 47.7% of males and 50.6% of females individually, while the macrocytic condition was evident in 18.3% of males and 12.75% of female subjects, examined on peripheral smear tests. A lower range of packed cell volume (PCV) was observed in 60.6% of males and 81% of females, indicative of a high risk of anemia and hypochromic microcytic RBCs. This may be due to decreased protein intake and nutritional deficiencies among the elderly.

India comprises 9% of the elderly population, and it is evident that aging leads to multiple health issues and morbidities [21], this study divulged the co-morbid conditions affecting the Indian elderly population majorly: generalized weakness (28.4%), followed by CVD (6.5%), diabetes mellitus (22.0%), HTN (6.4%), as compared to other studies chronic obstructive pulmonary disease (37%), diabetes mellitus (47%), and HTN (49%) are the most common conditions.

This study addressed the crucial topic of healthy aging and its effects on the elderly population. In addition to highlighting deviations in hematological parameters from normal values and their correlations with a variety of health issues, the relationship between hematological parameters and common health conditions in the elderly can aid in the development of stringent healthcare strategies. Healthcare practitioners can use these data as a useful indicator to recognize and address health risks in the elderly population in future studies and investigations. The study offers insights into clinical practice in the real world and its applicability to the provision of healthcare.

Along with practical perception, this study had some limitations, such as the small sample size of 188 patients from a single center, meaning that the results did not accurately reflect the diversity of the geriatric population as a whole. Due to the cross-sectional nature of the study, a causal relationship could not be established. Red blood cell count, Hb, HCT, and peripheral smear results were the main subjects of the investigation; therefore, an extensive examination of additional hematological indicators that would paint a fuller picture of the participants' health was absent from this report. Changes in hematological markers or health conditions over a longer period of time could not be captured by the study because participants were not followed over time. Hence, a longitudinal search is required to study all the missing parameters.

## Conclusions

Overall, this study concluded that regular monitoring of hematological parameters in the geriatric population is necessary to ascertain the various risks associated with their health. The results indicated a significant deviation in normal hematological parameters, which were associated with various health issues. Hematological parameters are convenient, feasible, and time-saving for primary screening. These parametric associations can be used as risk indicators, which can help healthcare professionals ensure good health in the geriatric population.
